# The difference in scotopic and photopic pupil responses: a potential indicator for long-term glycemic management

**DOI:** 10.3389/fneur.2026.1785905

**Published:** 2026-06-17

**Authors:** Ye Li, Zeyu Pan, Qi Ren, Fan Shi, Pengxia Wan

**Affiliations:** Department of Ophthalmology, The First Affiliated Hospital, Sun Yat-sen University, Guangzhou, China

**Keywords:** autonomic neuropathy, diabetes mellitus, glycated hemoglobin, neurophysiology, pupil diameter, pupillometry

## Abstract

Diabetic autonomic neuropathy manifests in pupillary dynamics, often serving as a subtle precursor to systemic complications. Therefore, quantitative biomarkers that can effectively index disease chronicity and autonomic status—independent of glycemic control—are required for precise monitoring. Hence, we evaluated the diagnostic utility of the pupil diameter (PD) difference under varying lighting conditions in a cohort of diabetic patients and non-diabetic controls. We employed high-resolution pupillometry to quantify pupillary responsiveness and assessed the association between PD difference, glycated hemoglobin (HbA1c), and disease duration. We observed a significant attenuation in the PD difference in the diabetic group (0.94 ± 0.44) compared to controls (1.21 ± 0.48), with high statistical significance. Data from receiver operating characteristic (ROC) analysis suggested that PD difference meets the performance criteria for identifying established disease duration (>1 year), indicating that quantitative pupillometry provides a viable clinical endpoint for assessing the chronicity of diabetes and its associated autonomic neuropathy.

## 1 Introduction

Diabetic autonomic neuropathy is a frequent yet often underdiagnosed complication in the management of diabetes mellitus, a condition posing a growing global health challenge with a projected prevalence of 500 million individuals by 2030 (1–3). While diabetes is associated with severe long-term sequelae, including retinopathy and nephropathy, the assessment of autonomic dysfunction remains diagnostically challenging (4). Typically, screening for autonomic neuropathy relies on complex cardiovascular reflex tests or subjective symptom reporting, which may preclude early detection (5).

Pupillary function serves as a sensitive physiological proxy for the autonomic nervous system (6). Pupillary light reactivity could be modified by the sympathetic and parasympathetic dysregulation inherent to diabetes, often attributed to small-nerve fiber pathology (7–9). Consequently, diabetic patients frequently exhibit diminished pupillary dynamics. Unlike subjective clinical assessments, quantitative measurement of pupil diameter (PD) offers a potential standardized window into autonomic integrity (10).

Emerging data suggest that pupillary variables are altered in diabetic populations (11). However, systematic evaluations quantifying the specific association between pupil diameter, disease duration, and the extent of autonomic neuropathy are limited in the existing literature. Therefore, we conducted a comparative study between diabetic patients and non-diabetic controls, with the primary aim to assess the diagnostic value of pupil diameter as a quantitative biomarker for early diabetic autonomic neuropathy and to explore its potential to improve diabetes management strategies.

## 2 Methods

### 2.1 Study design and participants

The study was a prospective, observational clinical cohort study performed at the Department of Ophthalmology, The First Affiliated Hospital of Sun Yat-Sen University. The study protocol was approved by the Ethics Committee of the First Affiliated Hospital of Sun Yat-sen University (Approval No. [2025]ER75). Ethics committee approval was obtained prior to recruitment, and written informed consent was obtained from all participants according to local regulations. The study was performed in accordance with the Declaration of Helsinki.

Patients were screened for inclusion between October 2022 and June 2023. Patients were included if they were aged 18–80 years. Participants were stratified into two cohorts: a Diabetes Mellitus (DM) group (*n* = 133) and a non-diabetic Control group (*n* = 229). Patients were excluded if they had ocular or neurological conditions interfering with pupillary function; current use of systemic or topical medications affecting pupillary dynamics; anterior segment abnormalities, including iris or pupil defects or the presence of a relative afferent pupillary defect; or concurrent optic nerve or retinal pathologies other than diabetic retinopathy, as well as significant corneal or vitreous opacities.

Baseline demographics, fasting plasma glucose (FPG), and glycated hemoglobin (HbA1c) levels were recorded. A comprehensive ophthalmic examination was performed, including chromatic pupillometry using OPD Scan III aberrometer (NIDEK Co. Ltd., Gamagori, Japan). All measurements were conducted by a single experienced ophthalmologist who was blinded to the study grouping to minimize bias.

### 2.2 Pupil diameter measurement

Static pupillometry was performed using the OPD Scan III aberrometer (NIDEK Co. Ltd., Gamagori, Japan). Pupil diameter was measured under two predefined illumination conditions: photopic and scotopic (photopic (85 cd/m^2^)) and scotopic (0.01 cd/m^2^). Participants were instructed to fixate on a distant internal target to minimize accommodation-related variability. Measurements were obtained under standardized ambient conditions by the same experienced ophthalmologist, who was masked to group allocation. The PD difference was calculated as (12):

PDdifference=scotopicPD−photopicPD

For analysis, only right eye was included.

### 2.3 Data analysis

Statistical Analysis Multivariable logistic regression was done to model the association between PD difference and diabetes-related outcomes, specifically disease chronicity. Models were adjusted for potential confounders, including age, sex, and glycated hemoglobin (HbA1c) levels. In these models, PD difference was entered as the primary independent variable. Receiver operating characteristic (ROC) curve analyses were performed to assess the diagnostic value of PD difference compared with HbA1c. The area under the curve (AUC) was calculated, and optimal cutoff values were determined to quantify the predictive performance for the chronicity of diabetes.

## 3 Results

### 3.1 Sample characteristics

Table 1 displays the demographic information and clinical variables of the subjects included in this study. Overall, 362 participants were included in the present study.

**Table 1:** Characteristics of the subjects included in the study.

Characteristic	Overall *N* = 362^1^	DM *N* = 133^1^	Control *N* = 229^1^	*p*-value^2^
Gender				0.12
Male	158 (44%)	51 (38%)	107 (47%)	
Female	204 (56%)	82 (62%)	122 (53%)	
Age, year	68 (12)	68 (11)	68 (13)	0.9
FPG, mmol/L	6.11 (2.22)	6.93 (2.54)	5.20 (1.32)	<0.001
HbA1c, %	7.51 (1.42)	7.81 (1.32)	5.94 (0.76)	<0.001
Scotopic PD (mm)	4.59 (0.85)	4.31 (0.90)	4.74 (0.79)	<0.001
Photopic PD (mm)	3.46 (0.62)	3.38 (0.69)	3.50 (0.58)	0.10
RNFL, um				
TS	137 (32)	141 (28)	131 (37)	0.057
NS	109 (28)	113 (28)	102 (26)	0.008
NI	114 (33)	116 (35)	113 (30)	0.6
TI	147 (36)	144 (36)	151 (37)	0.2

^1^*n* (%); Mean (SD).

^2^Pearson's Chi-squared test; Welch Two Sample t-test; Fisher's exact test.

DM, diabetes mellitus; FPG, fasting plasma glucose; HbA1c, glycated hemoglobin; RNFL, retinal nerve fiber layer; T, temporal; N, nasal; S, superior; I, inferior, PD, pupil diameter. Combinations of letters indicate sectors (e.g., TS, temporal superior; NI, nasal inferior).

**Figure 1:**
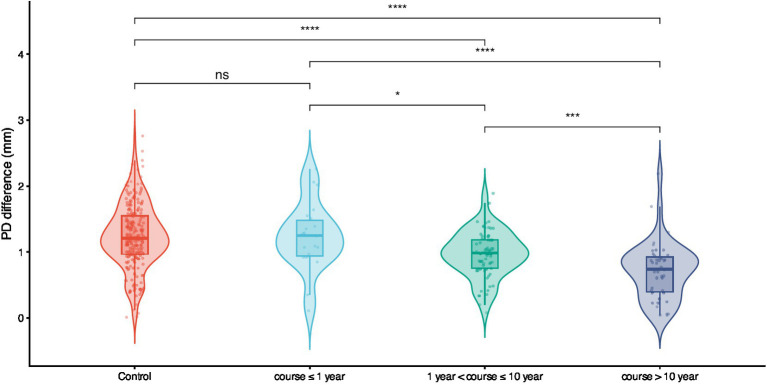
Comparison of pupil difference between DM and Control Group. Violin plots (showing distribution shape) are overlaid with box plots (median and interquartile ranges) for each group; individual data points are shown as dots. ****p < 0.0001, ***p < 0.001, **p < 0.01, *p < 0.05, ns *p* > 0.05.

The PD difference (the difference in PD between scotopic and photopic) was significantly lower in the DM group. Patients with a diabetes duration of ≤ 1 year showed no statistically significant difference compared to controls (*p* > 0.05). However, a significant decline was observed in patients with a duration between 1 and 10 years (*p* < 0.0001) and > 10 years (*p* < 0.0001) compared to controls. Notably, the decline was progressive: the 1–10 years group was significantly lower than the ≤ 1 year group (*p* < 0.05), and the > 10 years group demonstrated a significantly further reduction compared to the 1–10 years group (*p* < 0.01). These findings indicate a significant decrease in pupil sensitivity among patients with DM (Figure 1).

### 3.2 Diagnostic performance of pupillary diameter difference and HbA1c

**Figure 2:**
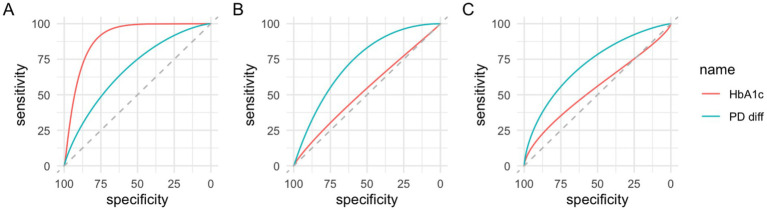
ROC curves comparing diagnostic performance of pupillary diameter difference and HbA1c. **(A)** DM diagnosis. **(B)** DM duration >1 year diagnosis. **(C)** DM duration >10 years diagnosis.

ROC curve analyses were performed to evaluate the diagnostic performance of PD difference and HbA1c across different clinical scenarios (Figure 2).

For DM diagnosis, HbA1c demonstrated excellent discriminatory ability with an effective AUC of 0.914, significantly outperforming PD difference which achieved an AUC of 0.698 (95% CI: 0.643–0.754) (*p* < 0.001, DeLong test). The optimal cut-off value for PD difference was 1.095, yielding sensitivity of 69.9% and specificity of 65.1%.

Diabetes Duration Assessment Among confirmed DM patients, PD difference showed superior performance in predicting disease chronicity. For identifying DM duration >1 year, PD difference achieved good discriminatory ability with an AUC of 0.736 (95% CI: 0.613–0.858), optimal cut-off of 1.065, sensitivity of 74.8%, and specificity of 68.2%. In contrast, HbA1c showed poor predictive performance with an AUC of 0.485 (95% CI: 0.370–0.601), indicating minimal discriminatory ability (*p* = 0.008, DeLong test). Similarly, for predicting DM duration >10 years, PD difference maintained good performance with an AUC of 0.731 (95% CI: 0.640–0.822), optimal cut-off of 0.975, sensitivity of 81.4%, and specificity of 58.9%. HbA1c continued to show poor discriminatory ability with an AUC of 0.435 (95% CI: 0.314–0.556), optimal cut-off of 6.85%, sensitivity of 30.2%, and specificity of 86.7% (*p* < 0.001, DeLong test).

## 4 Discussion

The principal finding of this study is that quantitative pupillary dynamics are significantly attenuated in patients with diabetes compared with non-diabetic controls. This reduction in the pupil diameter (PD) difference supports the hypothesis of early autonomic involvement in diabetes, likely attributable to the progressive degeneration of small nerve fibers induced by chronic hyperglycemia and metabolic dysregulation (13). The impaired pupillary reactivity observed under controlled photopic and scotopic conditions reflects a disruption in the delicate balance between sympathetic and parasympathetic regulation, providing a functional readout of the underlying neuropathy. In particular, impairment of the afferent limb of the pupillary light reflex—such as retinal damage from diabetic retinopathy or optic nerve dysfunction—may reduce the sensory input required to elicit normal pupillary responses. Additionally, local iris abnormalities, including ischemia or rubeosis iridis, may directly affect the contractile properties of the iris sphincter and dilator muscles, thereby limiting pupil reactivity. These factors could lead to an apparent reduction in PD difference independent of, or in combination with, autonomic neuropathy.

Crucially, our analysis revealed a strong inverse association between PD difference and the duration of diabetes. This suggests that pupillary dysfunction mirrors the chronicity of neurodegeneration. In the multivariable models adjusting for age, sex, and HbA1c, PD difference remained an independent predictor of disease status. A key insight from this study is the divergence between structural/functional nerve damage (measured by PD) and metabolic control (measured by HbA1c). While HbA1c remains the gold standard for monitoring glycaemic control, our data indicate it has limited utility in assessing established autonomic neuropathy. Conversely, PD difference provides distinct, additive associative information, serving as a specific biomarker for the neurological sequelae of diabetes.

The clinical implications of these findings are substantial. The ROC analysis demonstrated that the PD difference possesses high diagnostic utility for identifying long-standing disease and potential autonomic compromise. Currently, screening for diabetic autonomic neuropathy is often complex or subjective (14–17). Automated pupillometry offers a rapid, non-invasive, and objective alternative that could be integrated into routine outpatient workflows (18). By acting as a surrogate marker for systemic autonomic status, PD assessment could facilitate early risk stratification and prompt timely intervention before the onset of irreversible complications.

Our study has several limitations. First, the monocentric design and the specific demographics of our cohort, which exclusively included patients with Type 2 Diabetes Mellitus, may limit the generalizability of the findings to broader populations. Second, we did not explicitly quantify the burden of non-diabetic comorbidities, which could act as unmeasured confounders. Finally, the cross-sectional nature of the study precludes causal inferences regarding the temporal progression of pupillary dysfunction. Future longitudinal studies are needed to map the trajectory of pupillary dysfunction changes over time and to validate cut-off values for clinical decision-making.

In conclusion, this study establishes quantitative pupillometry as a robust and independent indicator of diabetic autonomic neuropathy. By capturing the functional decline of the autonomic nervous system, PD measurement complements traditional metabolic monitoring. These findings support the integration of pupillometry into the multimodal management of diabetes to improve the early detection and monitoring of neurodegenerative complications.

## Data Availability

The raw data supporting the conclusions of this article will be made available by the authors, without undue reservation.
